# Recurrence of chronic subdural hematoma due to low-grade infection

**DOI:** 10.3389/fneur.2022.1012255

**Published:** 2022-09-23

**Authors:** Daniel Dubinski, Sae-Yeon Won, Svorad Trnovec, Kseniya Gounko, Peter Baumgarten, Philipp Warnke, Daniel Cantré, Bedjan Behmanesh, Joshua D. Bernstock, Thomas M. Freiman, Florian Gessler, Steffen Sola

**Affiliations:** ^1^Department of Neurosurgery, Rostock University Medical Center, Rostock, Germany; ^2^Department of Neurosurgery, University Hospital, Schiller University Jena, Jena, Germany; ^3^Institute for Medical Microbiology, Virology and Hygiene, Rostock University Medical Center, Rostock, Germany; ^4^Institute of Diagnostic and Interventional Radiology, Pediatric Radiology and Neuroradiology, Rostock University Medical Center, Rostock, Germany; ^5^Department of Neurosurgery, Brigham and Women's Hospital, Harvard Medical School, Boston, MA, United States

**Keywords:** chronic subdural hematoma, dexamethasone, infected subdural hematoma recurrence, MMA occlusion, recurrence

## Abstract

Despite the high incidence and multitudes of operative techniques, the risk factors for chronic subdural hematoma (CSDH) recurrence are still under debate and a universal consensus on the pathophysiology is lacking. We hypothesized that clinically inapparent, a low-grade infection could be responsible for CSDH recurrence. This investigation is a single-center prospective observational study including patients with recurrent CSDH. In total, 44 patients with CSDH recurrence received an intraoperative swab-based microbiological test. The intraoperative swab revealed an inapparent low-grade hematoma infection in 29% of the recurrent CSDH cases. The majority (69%) of the identified germs belonged to the *staphylococcus* genus. We therefore, propose a novel potential pathophysiology for CSDH recurrence.

## Introduction

Chronic subdural hematoma (CSDH) is a common neurosurgical condition that affects primarily the elderly patient with an estimated incidence of up to 20 per 100,000 persons per year (1, 2). With the increasing age in the western population and the increasing use of anticoagulation, the incidence of CSDH is expected to be further rising in the near future (3). Despite a variety of established surgical techniques, such as burr-hole, twist drill, craniotomy with and without drain placement, or inner membrane tearing the optimal technique and postoperative management is still under debate (4, 5). One of the most challenging steps in the CSDH treatment is the high recurrence rate of up to 60% (6). Prediction models remain inconsistent with no implementation in clinical practice so far (7). Nevertheless, several risk factors for CSDH recurrence were described previously including the lack of postoperative drainage and operative technique (burr-hole vs. craniotomy) (6). Recent studies proposed a possible inflammatory component as the underlying pathophysiology for CSDH recurrence and several clinical approaches have targeted hematoma membrane inflammatory cascade including MMA embolization and dexamethasone treatment (8, 9). However, postoperative wound infections in CSDH are so far only known as case reports with fewer than 50 cases reported in the literature (10–15). We aimed to investigate the hypothesis of a possible subclinical low-grade hematoma infection as the driving force for CSDH recurrence.

## Methods

### Patients and data collection

The study was conducted according to the guidelines of the Declaration of Helsinki, and approved by the local Ethics Committee. Patients' consent was waived as this was a non-interventional study type. All patients that were admitted to the neurosurgical department of the authors' institution between August 2017 and June 2020 with the diagnosis of a CSDH were included in the analysis. Inclusion criteria were: (1) Patients with recurrent chronic subdural hematoma diagnosed by CT- or MRI-scan, (2) patients aged 18 years and above, and (3) patients with no wound infection. All patients with recurrent CSDH who required revision surgery received an intraoperative swab test. Patient characteristics and medical data were collected using the institutional electronic database. As this was a non-interventional monocentric study, patient consent was waived. Exclusion criteria included apparent postoperative wound infection, lack of radiological data, pre-existing hematological disorders, or hospital discharge in < 24 h after admission. Investigated medical record parameters included: age at ad-mission, sex, Glasgow Coma Scale (GCS) at admission, preoperative laboratory analysis, midline shift, surgical treatment, duration of surgery and drain placement, preexisting conditions, clinical course, and GCS at discharge.

### Surgical treatment

All patients received a perioperative 2 g cefazolin prophylaxis for both the initial evacuation and the revision surgery. A closed drainage system after evacuation was implanted in all cases. A postoperative CT scan was obtained prior to drain removal. Prophylactic low molecular weight heparin was started after 24 h in all patients. In cases with preoperative anticoagulation, the anticoagulant was re-administered not earlier than postoperative day 7. Recurrence was defined as the accumulation of chronic subdural fluid requiring re-operation. A second surgery performed during the same hospitalization was not considered a recurrence.

### Microbiological testing

Microorganisms were identified by matrix-assisted laser desorption/ionization time-of-flight mass spectrometry (MALDI-TOF-MS) with a Shimadzu/Kratos “AXIMA Assurance” MALDI-TOF mass spectrometer (Shimadzu Germany Ltd., Duisburg, Germany). For MALDI-TOF analyses, isolates were prepared using alpha-cyano-4-hydroxy cinnamic acid (bioMérieux, Marcy l'Etoile, France) as matrix. Spectral fingerprints were analysed using the Vitek MS IVD database version 3.2.0.-6. (bioMérieux, Marcy l'Etoile, France).

EUCAST based Antibiotic Susceptibility Testing (AST) was performed on the VITEK 2 platform using the appropriate VITEK 2 AST cards (bioMérieux, Marcy l'Étoile, France) according to manufacturer's instructions or by agar diffusion epsilometer testing according to EUCAST guidelines, if indicated. Routine culture comprised incubation on aerobic and anaerobic solid media (Columbia agar, MacConkey agar, Chocolate agar, Schaedler agar, Schaedler KV agar, Becton Dickinson GmbH, Heidelberg, Germany) as well as in liquid media (brain-heart infusion broth, thioglycollate broth, Becton Dickinson GmbH, Heidelberg, Germany), at 37°C and 5% CO_2_/20% O_2_ (CO_2_-enriched aerobic) or 0% O_2_/10% CO_2_/10% H2/80% N2 (anaerobic) atmosphere, respectively. CO_2_-enriched aerobic and anaerobic conditions were provided by employing the KB 115 (Binder GmbH, Tuttlingen, Germany) incubator and the Anoxomat III (Advanced Instruments, Norwood, MA, USA) with appropriate jars, respectively.

### Study design

The present analysis is a prospective, single-center observational study of patients with recurrent CSDH. The aims of the study were (1) to observe the incidence of germ detection in recurrent CSDH and (2) to correlate the germ detection with patient outcome.

### Statistics

Data analysis was performed with IBM SPSS Statistics Version 23.0 (SPSS Inc., IBM Corp., Armonk, NY, USA). For patient characteristics, descriptive statistics were used. Fisher's exact test was used for the comparison of categorical variables between the cohorts. For continuous parameters, the Wilcoxon/Mann–Whitney test was used. To assess the impact of the variables, odds ratios (*OR*s) with 95% confidence intervals (*CI*s) were calculated. Results with *p* ≤ 0.05 were considered statistically relevant.

## Results

### Participants and descriptive

#### Data

**Figure 1:**
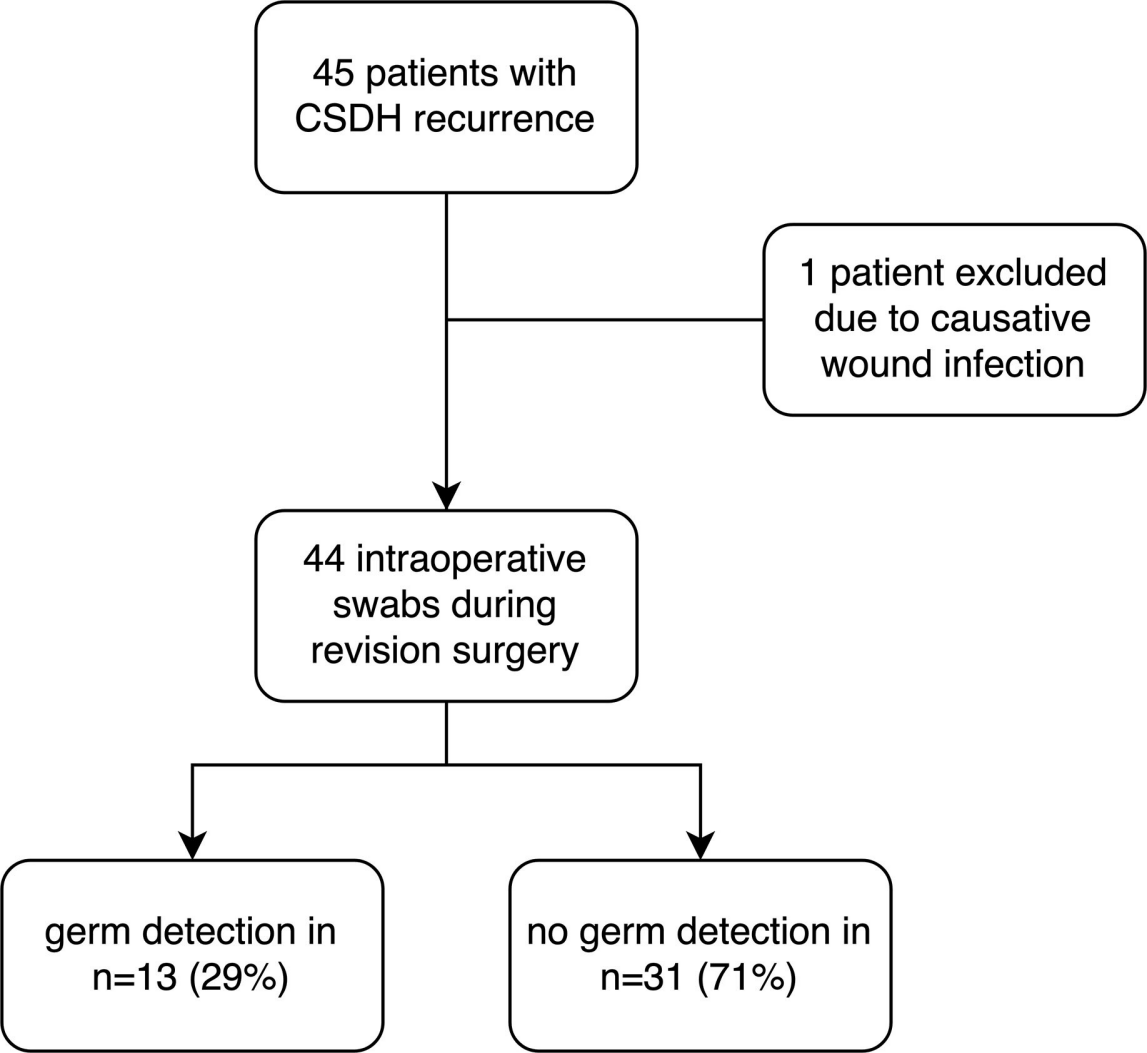
Study allocation with the illustration of the study protocol.

Of 141 patients with operative evacuation of CSDH, 45 patients showed recurrence that warranted revision surgery (31%). Indication for revision surgery was defined as an increase in the size of the residual hematoma or persisting neurological symptoms in association with a still existing, space-consuming hematoma residual. One patient was excluded due to a postoperative wound infection according to the exclusion criteria. Hence, 44 patients who underwent revision surgery for recurrent CSDH were included (Figure 1). The average age was 76.5 (interquartile range [IQR]: 69–81) and 35 (80%) of the patients were men. The median GCS at admission was 14.5 (IQR: 14–15) and 29 patients (66%) received anticoagulation therapy. The median C-reactive protein (CRP) at admission was 8.5 mg/l (IQR: 3–20.5) and median leukocyte count was 8.2 109/L (IQR: 6.8–9.4). In addition, 30 patients (68%) received burr-hole hematoma evacuation vs. 14 patients (32%) that received mini-craniotomy. In terms of preexisting comorbidities, 9 patients (20%) had arterial fibrillation, 28 patients (64%) hypertension, 14 (32%) diabetes, and 12 patients (27%) coronary heart disease. During the clinical course, one patient developed pneumonia (2%) and three patients urinary tract infection (7%). The median GCS at discharge was 15 (IQR 14.3–15) (Table 1).

**Table 1:** Demographics, management, and surgical data.

**Patient characteristics**	***N* = 44**
Sex	
male, n (%)	35 (80)
Age, mean (IQR)	76.5 (69–81)
Admission status	
GCS at admission, median (IQR)	14.5 (14–15)
CRP at admission, median mg/l (IQR)	8.5 (3–20.5)
Leukocytes at admission, 10E9/L (IQR)	8.2 (6.8–9.4)
Anticoagulation at admission, n (%)	29 (66)
Midline-shift, mm (IQR)	8 (5.5–12)
Neurosurgical approach	
Burrhole, n (%)	30 (68)
Duration of surgery, minutes (IQR)	36 (22.5)
Duration of drainage, days (IQR)	3 (3–4)
Preexisting conditions	
Atrial fibrillation, n (%)	9 (20)
Hypertension, n (%)	28 (64)
Diabetes, n (%)	14 (32)
Coronary heart disease, n (%)	12 (27)
Clinical course	
Pneumonia, n (%)	1 (2)
Urinary tract infection, n (%)	3 (7)
Antibiotic usage	4 (9)
Discharge status	
GCS at discharge, median (IQR)	15 (14.3–15)

#### Characteristics and admission status and inapparent germ detection in recurrent CSDH

We found no significant association between sex and germ detection in patients with recurrent CSDH (69% male in the case cohort vs. 84% in the control group; *p* = 0.24). Among the 44 patients with CSDH recurrence, patients with germ detection presented with significantly increased CRP at re-admission when compared to patients with recurrent CSDH and without germ detection (*p* = 0.004). However, leukocyte counts did not show significant differences between the two groups (*p* = 0.34). Further, patients in the hematoma germ detection cohort had significantly reduced GCS admission status when compared with the control cohort (*p* = 0.002). The presence of anticoagulation at admission and the mid-line-shift were not associated with germ detection (Table 2).

**Table 2:** Analysis of juxtaposed characteristics according to inapparent germ detection in chronic subdural hematoma (CSDH).

**Patient characteristics (*N* = 44)**	**Germ detection**				
	**yes (n = 13)**	**no (n = 31)**	**OR**	**95% CI**	***P* value**
Sex					
male, n (%)	9 (69)	26 (84)	0.43	0.09–1.97	0.24
Age, mean (IQR)	75 (70–80)	78 (68.5–83)	n/a	5.67–11.67	0.48
Admission status					
GCS at admission, median (IQR)	14 (14–15)	15 (14–15)	n/a	0.36–1.64	0.002
CRP at admission, mg/l (IQR)	10.7 (3.4–21.8)	6.4 (2.7–20)	n/a	1.42–7.17	0.004
Leukocytes at admission, 10E9/L (IQR)	8.7 (2.3)	7.8 (6.3–9.8)	n/a	1.06–2.80	0.34
Midline-shift, mm (IQR)	8 (7–13)	7.5 (4–11)	n/a	2.83–3.84	0.76
Anticoagulation at admission, n (%)	9 (69)	20 (65)	1.23	0.30–4.96	1
Neurosurgical approach					
Burrhole, n (%)	8 (62)	22 (71)	0.65	0.16–2.55	0.72
Duration of surgery, minutes (IQR)	39 (28–51)	33 (24–46)	n/a	9.31–25.31	0.35
Duration of drainage, days (IQR)	3 (3–4)	3 (3–3.5)	n/a	1.41–1.64	1
Preexisting conditions					
Atrial fibrillation, n (%)	3 (32)	6 (19)	1.25	0.26–5.99	1
Hypertension, n (%)	8 (62)	20 (65)	0.88	0.23–3.35	1
Diabetes, n (%)	5 (38)	9 (29)	1.52	0.39–5.95	0.72
Coronary heart disease, n (%)	4 (31)	8 (26)	1.27	0.30–5.32	1
Clinical course					
Pneumonia, n (%)	1 (3)	0 (0)	n/a	n/a	n/a
Urinary tract infection, n (%)	2 (15)	1 (3)	n/a	0.44–66.31	0.24
Discharge status					
GCS at discharge, median (IQR)	15 (13.7–15)	15 (15–15)	n/a	1.45–1.45	1

### Neurosurgical approach and inapparent germ detection in recurrent CSDH

Among the included 44 patients with CSDH recurrence, eight patients (62%) with germ detection received burr-hole evacuation for hematoma evacuation vs. 22 patients (71%) in the control group which was not statistically significant (*p* = 0.72). Furthermore, the duration of drainage was not associated with germ detection in recurrent CSDH (*p* = 1). The duration of surgery was also not associated with germ detection in recurrent CSDH (*p* = 0.35).

### Preexisting conditions and inapparent germ detection in recurrent CSDH

In our analysis, three patients (32%) with recurrent CSDH and germ detection had arterial fibrillation compared with the six patients (16) in the control cohort (*p* = 1). None of the other analyzed preconditions were associated with the germ detection in recurrent CSDH (hypertension *p* = 1; diabetes mellitus *p* = 0.72, and coronary heart disease *p* = 1).

### Clinical course, status at discharge, and inapparent germ detection in recurrent CSDH

In our study, the development of pneumonia during the clinical course was not associated with germ detection in recurrent CSDH with 1 patient in the case group vs. 0 patients in the negative cohort. Furthermore, two patients with germ detection (15%) developed urinary infection during the clinical course vs. 1 (3%) in the control group (*p* = 0.24). Postoperative urinary infection was therefore not associated with germ detection. Patients with inapparent germ detection had a median GCS score of 15 (IQR 14–15). The median GCS score of patients with recurrent CSDH in the control group was also 15 (IQR 14–15). The GCS at discharge was therefore not associated with inapparent germ detection in CSDH.

### Composition of the germ detection in recurrent CSDH

**Figure 2:**
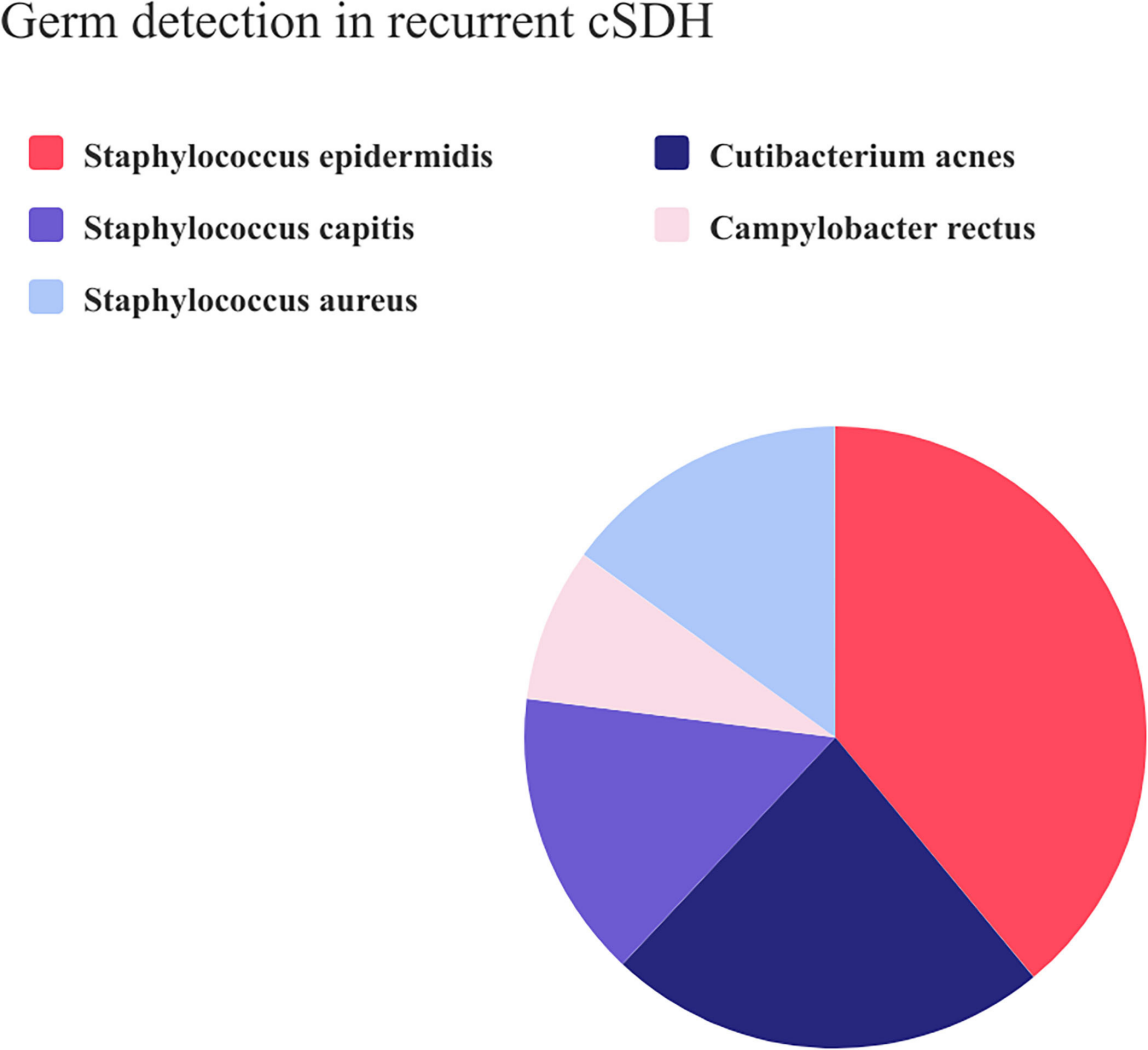
Pie chart composition of the positive microbiological swab results in recurrent CSDH.

Among the 13 patients with positive swab results, 39% were *Staphylococcus epidermidis*, 23% *Cutibacterium acnes*, 15% *Staphylococcus aureus*, 15% *Staphylococcus capitis*, and 8% *Campylobacter rectus* (Figure 2). Regarding the infectious etiological information of patients with pneumonia and urinary tract infection, we found different germ spectrum compared with the intracranial swab: two patients with UTI during their clinical course had *Enterococcus faecalis* and one patient had *Aerococcus urinae*. The patients with pneumonia had *Klebsiella pneumonia* as the causal germ.

## Discussion

Our study revealed that nearly 30% of patients with recurrent CSDH showed positive swab-based microbiological analyses at the time of revision surgery. We, therefore, propose low-grade infection as a potential contributor to CSDH recurrence. Among the identified germs, the majority belonged to the genus *Staphylococcus*. Further, two statistically significant risk factors for CSDH germ inoculation were identified.

The pathophysiology of CSDH formation is historically described as a consequence of subclinical trauma to bridging veins, which leads to subdural hemorrhagic fluid collection. These veins are especially vulnerable in patients with brain atrophy—a circumstance that could explain the increased incidence in older patients (17). However, this historical explanation is challenged nowadays. Several authors proposed a posttraumatic release of reactive inflammatory mediators from the hematoma membrane, such as fibrin, angiopoetin-2, vascular endothelial growth factor (VEGF), and several other cytokines (18, 19). The cascade proceeds to the formation of fragile *de novo* vessels, which leads to continuous exudation and hematoma expansion. This process is believed to take place during an asymptomatic latency period, which eventually transforms into the symptomatic period once the intracranial vault is exhausted (16).

The summarized pathophysiology could explain the effectiveness of middle meningeal artery embolization that has been reported in clinical trials. The de-vascularization of the hematoma membrane may lead to a reduction of the influx of inflammatory mediators from the hematoma membrane and consequently reduces the exudate, as has been verified in several clinical trials (9, 20), The reactive inflammatory cascade has also been addressed in medical treatment approaches, such as treatment with dexamethasone. Several retrospective studies did in fact show a reduction in CSDH recurrence upon steroid administration (21). However, a multicenter randomized trial led by Hutchinson et al. in 2020 showed conflicting results as dexamethasone treatment after CSDH evacuation led to more adverse events but fewer revision surgeries (8).

On the other hand, infections of CSDH are described as highly unusual, with fewer than 50 case reports available (10, 14, 22, 23). According to the available data, infected subdural hematoma is most commonly diagnosed in people aged 60 years and older who have immunologic dysfunction or an underlying disease, such as diabetes or chronic infection (11, 12). However, to the best of our knowledge, no equivalent investigation or a systematic analysis regarding a possible association between the CSDH recurrence and a low-grade infection has been performed so far.

In our analysis, roughly a third of the analyzed microbiological swabs in clinical inapparent patients with recurrent CSDH showed a positive test result. This finding is rather interesting and adds novel explanatory approaches to the held view of the aseptic inflammatory cascade. Chronic infection limited to the zone of a lesion is commonly described as a low-grade infection presenting without obvious local and systemic clinical signs of infection, as it is the case in our cohort (24). Our data indicate that a substantial part of recurrent CSDH is caused by an undiagnosed low-grade infection. The slightly increased and statistically relevant CRP level in our cohort could be an indication for the ongoing low-grade infection.

Since our investigation is pioneering the field of low-grade infection in CSDH, germ spectrum comparison with previous reports is difficult. A recent review of subdural empyema identified forty-seven cases with *Escherichia coli* in 13 cases (27%), *Salmonella* species in eight cases (17%), *Staphylococcus aureus* in six cases (13%), and *Streptococcus* species in five cases (10%). Four cases demonstrated *Klebsiella* spp. and *Campylobacter fetus* (9%) (10). In contrast, our cohort showed the genus *Staphylococcus* as dominant, which could indicate a consistently different pathophysiology. On the other hand, a recent analysis on causative low-grade infection in patients with screw loosening described a similar germ composition as in our cohort with the majority of the genus *Staphylococcus* (24). Whether this is an intraoperative contamination, surgical site infection or a hematogenous spread should be clarified in further studies. Furthermore, we have not obtained intraoperative swab-based microbiological testing for primary CSDH and hence cannot exclude the presence of a low-grade infection in this case. To answer this question, future studies should implement intraoperative swab-based microbiological testing for primary CSDH cases as well. Furthermore, due to clinically inapparent sings of infection, we have not obtained procalcitonin (PCT) values in this cohort. PCT could potentially reveal inapparent signs of infection and therefore should be included in the blood sampling of future studies.

The obvious limitation of this clinical study is the small sample size. We, therefore, cannot exclude underdiagnoses. As this is an observational study, confounding, selection bias, and uncontrolled statistical error risk cannot be excluded. However, further prospective randomized trials with large cohorts are necessary to validate our findings.

## Conclusion

According to our findings, clinically inapparent, low-grade infections in patients with recurrent CSDH may be observed during revision surgery. We therefore stress the necessity of intraoperative swab-based microbiological testing in patients with recurrent CSDH.

## Data availability statement

The raw data supporting the conclusions of this article will be made available by the authors, without undue reservation.

## Ethics statement

The studies involving human participants were reviewed and approved by Ethics Committee, University of Rostock and Medical Center. Written informed consent for participation was not required for this study in accordance with the national legislation and the institutional requirements.

## Author contributions

DD collected the data and wrote the first draft. SS initiated the swab testing. FG supervised the manuscript. All authors supplied additional information, edited the manuscript, contributed to critical review, and revision of the manuscript. All authors contributed to the article and approved the submitted version.

## Conflict of interest

Author DD received financial support from Novartis, Fresenius, Inovitro, and Novocure. None of these entities were involved in the study design, collection, analysis, interpretation of data, the writing of this article or the decision to submit it for publication. The remaining authors declare that the research was conducted in the absence of any commercial or financial relationships that could be construed as a potential conflict of interest.

## Publisher's note

All claims expressed in this article are solely those of the authors and do not necessarily represent those of their affiliated organizations, or those of the publisher, the editors and the reviewers. Any product that may be evaluated in this article, or claim that may be made by its manufacturer, is not guaranteed or endorsed by the publisher.
